# Bioactive Compounds from Edible and Medicinal Mushrooms in Obesity–Depression Comorbidity: Gut–Brain Axis Mechanisms and Functional Food Perspectives

**DOI:** 10.3390/foods15183265

**Published:** 2026-09-16

**Authors:** Wang Li, Dongze Chi, Xingmin Hu, Yuan Liu, Zhan Li, You Li, Yueting Dai, Yu Li

**Affiliations:** 1 Engineering Research Center of Edible and Medicinal Fungi, Ministry of Education, Jilin Agricultural University, Changchun 130118, China; lw08271006@163.com (W.L.); huxingm0528@163.com (X.H.); 17790099278@163.com (Y.L.); 2 Jilin Provincial Drug Review and Inspection Center, Changchun 130000, China; 792661309@163.com; 3 Chengde Academy of Agriculture and Forestry Sciences, Chengde 067000, China; m17701299082@163.com; 4 College of Chemistry and Life Sciences, Changchun University of Technology, Changchun 130012, China; lizhan0827@163.com

**Keywords:** microbiota, neuroinflammation, polysaccharides, nutraceutical development, bioactive compounds, gut–brain interactions

## Abstract

Obesity–depression comorbidity involves interconnected metabolic dysfunction, chronic low-grade inflammation, oxidative stress, neuroendocrine dysregulation, gut microbial dysbiosis, and impaired gut–brain communication. Edible and medicinal mushrooms provide nutrients and diverse bioactive compounds, including polysaccharides, terpenoids, nucleosides, sterols, peptides, and phenolic compounds. This narrative review integrates evidence on representative mushroom species, cultivation and fermentation factors affecting their composition, major bioactive compounds and biosynthetic pathways, gut–brain axis-related mechanisms, and opportunities for functional food development. Evidence derived predominantly from in vitro and animal studies suggests that mushroom-derived bioactive compounds may modulate gut microbial composition, intestinal barrier integrity, inflammatory and oxidative pathways, lipid and glucose metabolism, and neuroendocrine signaling. An exploratory database-based analysis further mapped predicted candidate molecular targets associated with inflammatory, metabolic, and neural processes. From a food science perspective, the compositional diversity of mushrooms and their compatibility with cultivation, fermentation, extraction, and formulation provide opportunities for developing conventional foods, functional foods, nutraceuticals, and standardized products. Overall, edible and medicinal mushrooms show promise as food and nutraceutical resources, but their translation into evidence-based functional products requires improved standardization, safety and bioavailability assessments, and well-designed human trials.

## 1. Introduction

Obesity and depression are prevalent and disabling conditions that frequently coexist. In 2021, approximately 2.11 billion adults aged 25 years or older were living with overweight or obesity, and this number is projected to reach 3.80 billion by 2050 [1]. The Global Burden of Disease Study 2021 estimated approximately 332 million prevalent cases of depressive disorders worldwide [2], while a subsequent forecasting analysis projected that this number could exceed 466 million by 2040 [3]. Their co-occurrence is clinically important because metabolic and psychological symptoms may reinforce one another through behavioral, pharmacological, and biological pathways, thereby complicating long-term management.

The relationship between obesity and depression involves several interconnected processes rather than a single causal pathway. Proposed shared mechanisms include chronic low-grade inflammation, insulin resistance, oxidative and mitochondrial stress, hypothalamic–pituitary–adrenal axis dysregulation, altered neuroendocrine signaling, intestinal barrier dysfunction, and gut microbial dysbiosis [4,5,6]. The microbiota–gut–brain axis provides a framework through which microbial metabolites, immune mediators, endocrine signals, and neural pathways may connect metabolic homeostasis with mood-related processes [5]. Current management relies on dietary and behavioral interventions and condition-specific pharmacotherapy, with bariatric surgery considered for eligible patients with obesity. However, evidence for nutritional interventions specifically addressing obesity–depression comorbidity remains heterogeneous [7], and some antidepressants are associated with modest differences in weight change [8]. These limitations support the investigation of food-based adjunctive strategies that can be incorporated into long-term dietary management, without assuming that such strategies can replace established clinical treatment.

Edible and medicinal mushrooms are nutrient-rich food resources containing proteins, dietary fibers, polysaccharides, terpenoids, nucleosides, sterols, peptides, and phenolic compounds [9,10]. Their compositional diversity and amenability to cultivation, fermentation, extraction, and food formulation support their use in conventional foods and their development as functional foods, nutraceutical ingredients, and standardized products. Evidence derived predominantly from in vitro and animal studies suggests that selected mushroom-derived compounds may influence gut microbial composition, intestinal barrier integrity, inflammatory and oxidative pathways, glucose and lipid metabolism, and neuroendocrine signaling [10,11,12]. Mushroom polysaccharides are of particular interest because their interaction with the gut microbiota may affect short-chain fatty acid production and host metabolic and immune responses [11]. Nevertheless, direct clinical evidence in individuals with obesity–depression comorbidity remains scarce, and the composition, bioavailability, safety, and effective dose of mushroom preparations require further characterization.

This structured narrative review integrates evidence on representative edible and medicinal mushroom species; cultivation and fermentation factors affecting their composition; major nutritional and bioactive compounds and their biosynthetic pathways; and mechanisms potentially relevant to obesity–depression comorbidity. Building on previous reviews of the antidepressant and microbiota-related effects of mushroom-derived compounds [10,11,12], the present review focuses specifically on the convergence of metabolic dysfunction, neuroinflammation, and microbiota–gut–brain communication in obesity–depression comorbidity. An exploratory database-based target-association analysis is also included to map candidate molecular targets and pathways and generate hypotheses for future experimental investigation. Finally, this review highlights the potential of edible and medicinal mushrooms as versatile resources for functional food and nutraceutical development and discusses future directions for translating their nutritional and bioactive properties into standardized, safe, and evidence-supported products.

## 2. Literature Search and Analytical Methodology

### 2.1. Literature Search and Evidence Synthesis

This work was designed as a structured narrative review, with its search strategy, scientific reasoning, and presentation developed with reference to the Scale for the Assessment of Narrative Review Articles (SANRA) quality domains [13]. PubMed, Web of Science Core Collection, and China National Knowledge Infrastructure (CNKI) were searched from database inception to 4 September 2026 using Boolean combinations of (“edible mushrooms” OR “medicinal mushrooms”) AND (obesity OR depression OR “bioactive compounds” OR cultivation OR fermentation OR “nutritional composition” OR “functional foods” OR “gut microbiota” OR “gut-brain axis”); equivalent Chinese terms were used for CNKI. Peer-reviewed original studies and relevant reviews reporting mushroom composition, biological activities, mechanistic evidence, or food applications were considered. Duplicate, non-peer-reviewed, irrelevant, and insufficiently documented records were excluded. Human evidence was prioritized, while animal and in vitro studies were used to interpret potential mechanisms. Owing to substantial heterogeneity in species, preparations, doses, and outcomes, the evidence was synthesized narratively rather than quantitatively.

### 2.2. Target-Association and Network Analysis

Six structurally defined mushroom-derived compounds—ganoderic acid A, antroquinonol, antrocin, erinacine A, cordycepin, and pachymic acid—were analyzed. Their molecular structures and canonical Simplified Molecular Input Line Entry System (SMILES) strings were obtained from PubChem. Computationally predicted candidate molecular targets were retrieved using SwissTargetPrediction, with the species restricted to *Homo sapiens*. Obesity- and depression-associated genes were retrieved separately from GeneCards using “obesity” and “depression” as the respective search terms and were subsequently used for downstream analysis. Gene names were standardized to official human gene symbols, and duplicate and unmapped records were removed. Venny 2.1 was used to identify the intersection between the obesity- and depression-associated gene sets, which was defined as the obesity–depression comorbidity-associated gene set. The non-redundant union of the candidate targets predicted for the six compounds was then generated, and its intersection with the comorbidity-associated gene set was determined. Intersections between each compound-specific candidate target set and the comorbidity-associated gene set were also calculated separately.

The resulting common candidate molecular targets were submitted separately and collectively to Search Tool for the Retrieval of Interacting Genes/Proteins (STRING) v12.0, with the species set to Homo sapiens, a minimum required interaction score of 0.400 (medium confidence), and the default active evidence channels. The resulting protein-association networks were visualized and analyzed using Cytoscape v3.10.2. Degree, betweenness centrality, and closeness centrality were calculated for each original network. Candidate hub nodes were generally retained when all three values were equal to or greater than their corresponding network-specific means using an AND rule. For the erinacine A network, degree and betweenness centrality were used because no nodes satisfied the simultaneous three-metric criterion. Comparable target-prediction and network-analysis workflows have been reported previously [14,15]. PubChem, SwissTargetPrediction, GeneCards, and STRING were accessed on 4 September 2026.

## 3. Resource Forms and Ecological Characteristics of Edible and Medicinal Mushrooms

### 3.1. Commercially Utilized Fungal Materials

Edible and medicinal mushroom products are derived from several biologically distinct materials, including fruiting bodies, cultured mycelia, sclerotia, and natural fungus–insect complexes. *Ganoderma lucidum*, *Lentinula edodes*, *Hericium erinaceus*, and *Grifola frondosa* are commonly utilized as fruiting bodies or fruiting-body extracts [16], whereas *Poria cocos* is used primarily as a sclerotium [17]. Resources associated with *Ophiocordyceps sinensis* include the naturally occurring fungus–insect complex and cultured mycelial preparations [18]. Because fungal species, tissue type, developmental stage, substrate, and cultivation conditions can influence nutritional and bioactive composition, the biological origin and preparation of each material should be specified when comparing composition, biological evidence, and food applications.

### 3.2. Ecological Groups and Habitat Constraints

**Figure 1:**
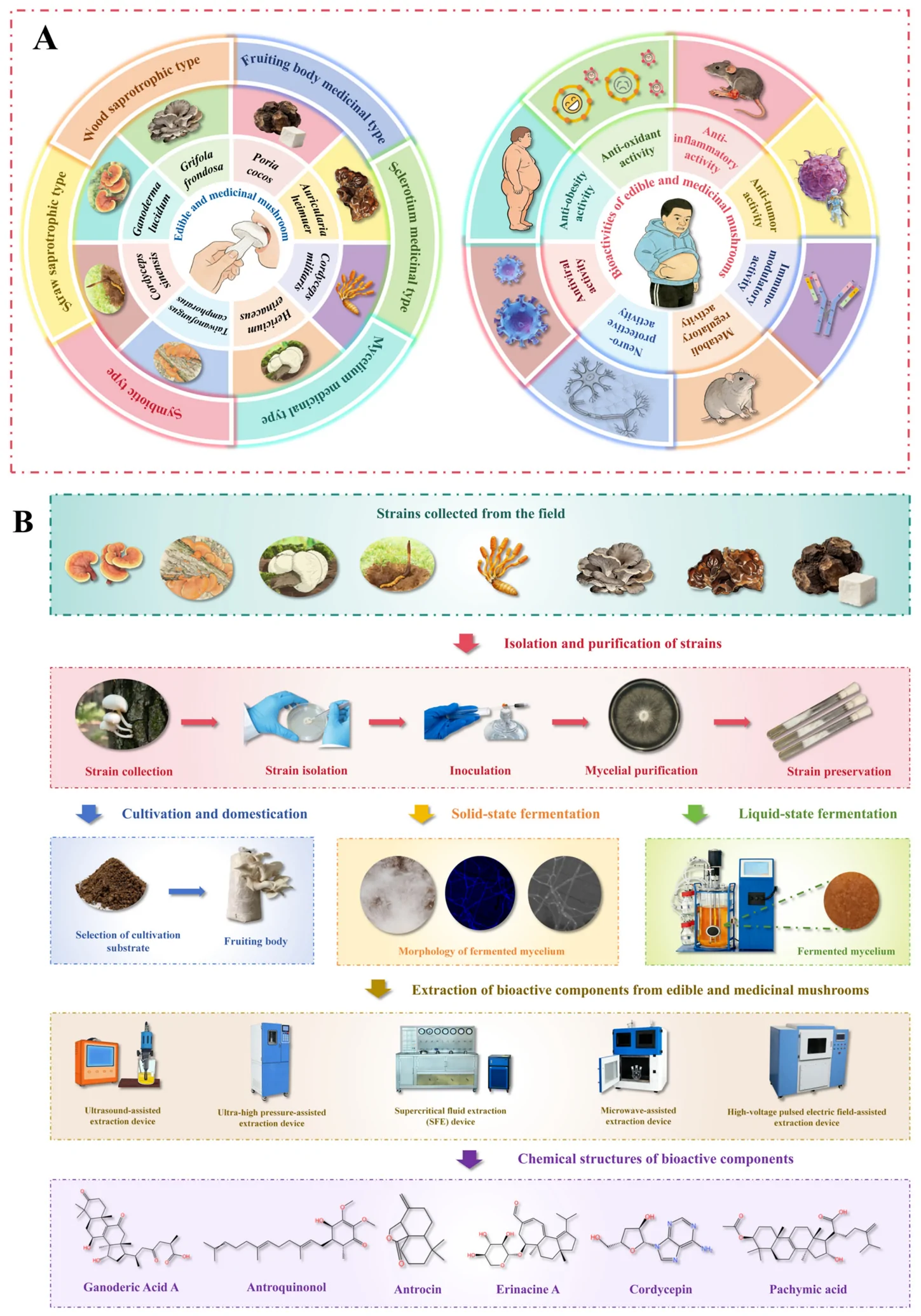
Overview of representative edible and medicinal mushroom resources, reported biological activities, and processing pathways. (**A**) Representative mushroom resources and biological activities reported in the literature; most functional evidence is derived from preclinical studies. (**B**) Schematic workflow encompassing field collection, strain isolation and preservation, cultivation or solid- and liquid-state fermentation, extraction, and representative mushroom-derived compounds.

Ecologically, the mushrooms discussed in this review represent several distinct resource groups. Saprotrophic wood-associated fungi include *G. lucidum*, *L. edodes*, *H. erinaceus*, *G. frondosa*, *Auricularia heimuer*, and *P. cocos*, whereas *Volvariella volvacea* is adapted to lignocellulosic agricultural residues such as rice straw [19]. *Tricholoma matsutake* is an ectomycorrhizal fungus associated mainly with conifer roots, which has limited the development of stable artificial cultivation systems [20]. The insect-associated group includes *O. sinensis* and *Cordyceps militaris* [18]. *Taiwanofungus camphoratus* is closely associated with the endemic tree *Cinnamomum kanehirae* in Taiwan [21], while wild *O. sinensis* occurs predominantly in alpine habitats of the Qinghai–Tibet Plateau and adjacent regions [22]. These ecological differences affect substrate selection, cultivation feasibility, resource conservation, and the reproducibility of mushroom-derived food ingredients (Figure 1A).

## 4. Cultivation and Fermentation Strategies and Compositional Variation

Production conditions are important sources of variation in the composition and reproducibility of mushroom-derived materials. Depending on fungal species and material type, production systems include log- and substrate-based fruiting-body cultivation, controlled industrial cultivation, semi-artificial cultivation, solid-state fermentation (SSF), and liquid-state fermentation (LSF). These systems differ in substrate, temperature, pH, aeration, light exposure, and harvest stage, which may affect biomass yield and the accumulation of nutrients and bioactive compounds. Because fruiting bodies, cultured mycelia, sclerotia, and fermented preparations are not compositionally interchangeable, their biological origin and production conditions should be specified when evaluating food applications and functional potential. The principal production routes and representative conditions are summarized in Figure 1B and Table 1.

### 4.1. Cultivation and Fermentation Modes

Table 1 summarizes representative cultivation and fermentation modes and reported culture conditions for eight edible and medicinal mushrooms. Their production systems vary according to species and the type of fungal material obtained, including fruiting bodies, cultured mycelia, sclerotia, and fungus–insect complexes. Fruiting-body production commonly involves log, bag, bottle, substrate-based, or controlled industrial cultivation, whereas *P. cocos* is primarily cultivated for sclerotial production and *O. sinensis* may be obtained through wild harvesting or semi-artificial cultivation. SSF and LSF provide additional approaches for producing fungal biomass and selected bioactive compounds. For example, bidirectional SSF of highland barley with *G. lucidum* increased triterpenoid content by 76.57%, while Tween 80 supplementation promoted mycelial biomass and exopolysaccharide production. Condition-specific metabolic regulation, including rotenone supplementation in *C. militaris*, has also been reported to increase cordycepin production under LSF conditions.

The eight edible and medicinal mushrooms exhibit distinct species-specific growth characteristics. Mycelial growth is optimal at 18–30 °C, with a slightly lower temperature for fruiting-body development. Relative humidity is 50–70% for mycelia and 60–95% for fruiting bodies or sclerotia. The suitable pH is mostly 5.0–6.8 (slightly acidic), except for *A. heimuer* (5.5–7.5). Most species require low diffused light (e.g., 300 lx for *G. lucidum*, 100–250 lx for *C. militaris*), while *P. cocos* grows in full darkness. For *T. camphoratus*, red light at 300 lx has been reported as a suitable illumination condition for cultivation.

### 4.2. Nutritional Components

**Figure 2:**
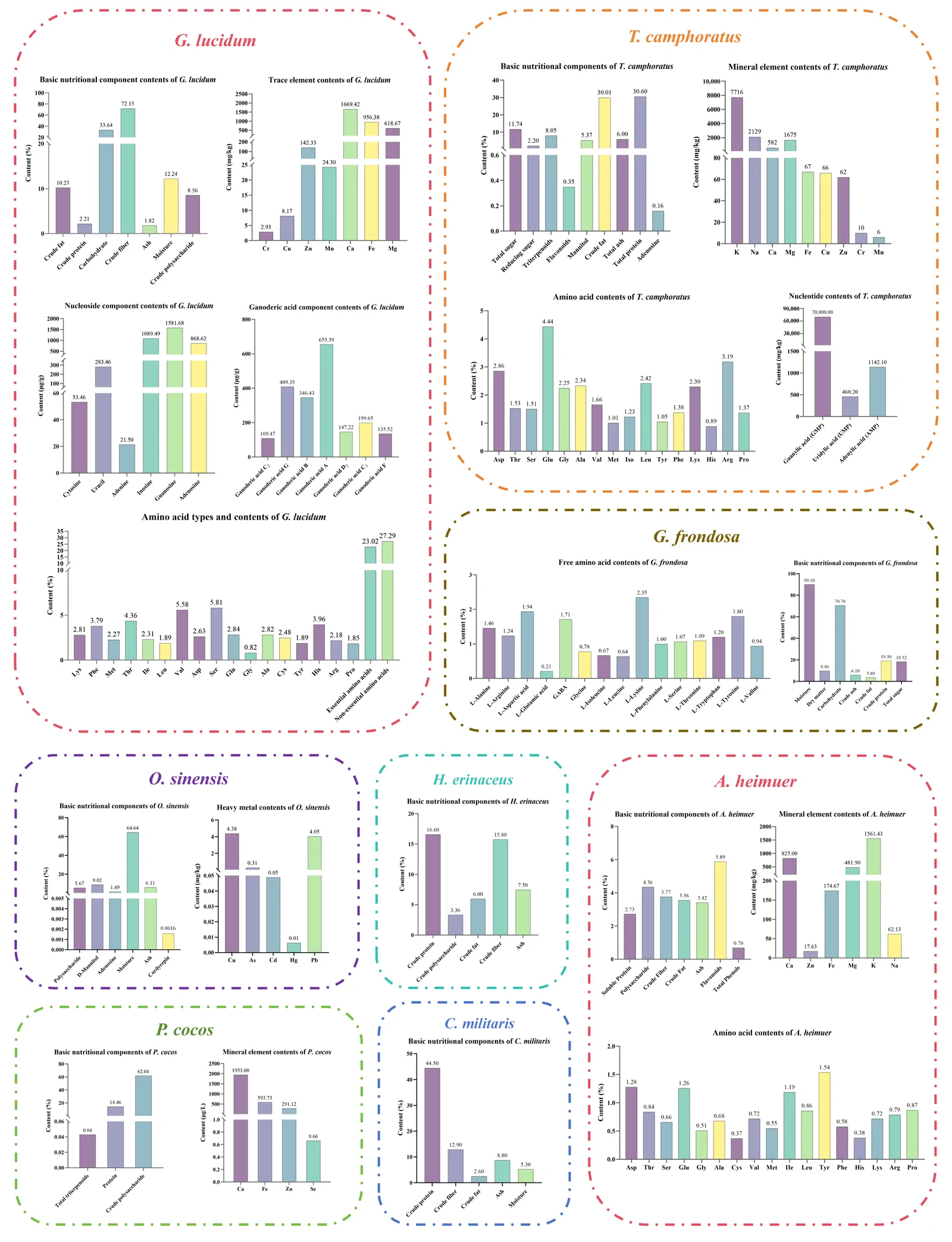
Nutritional, bioactive, amino acid, and elemental composition profiles of eight representative edible and medicinal mushrooms. The figure was created by the authors using data compiled from published studies [66,67,68,69,70,71,72,73,74]. Data are organized by species and component category, with the corresponding measurement units indicated in each panel.

The reported contents of nutritional and bioactive components vary among edible and medicinal mushrooms and across cultivation and fermentation systems. These data provide a compositional basis for characterizing different fungal materials and evaluating their potential food and functional applications. Representative compositional profiles are summarized in Figure 2.

Several edible and medicinal mushrooms exhibit notable dietary fiber and protein contents. The reported crude fiber contents of *G. lucidum* and *H. erinaceus* are 72.15% and 15.80%, respectively, whereas the reported crude protein contents of *C. militaris* and *H. erinaceus* are 44.50% and 16.60%, respectively. Representative species also display diverse amino acid profiles. *G. lucidum* contains 5.81 g/100 g serine and 5.58 g/100 g valine, while *T. camphoratus* contains 4.44% glutamic acid and 3.19% arginine. In the reported free amino acid profile of G. frondosa, L-lysine, L-aspartic acid, and L-tyrosine are present at 2.35%, 1.94%, and 1.80%, respectively. In animal studies, dietary fiber preparations derived from *H. erinaceus* and *Flammulina velutipes* have been reported to modulate lipid-related indices and metabolic pathways under hyperlipidemic or obese conditions [57,58]. Mushroom proteins are also being investigated for their nutritional quality and food-related functional properties [59]. These findings support the nutritional and functional potential of mushroom-derived fiber and protein.

Reported carbohydrate, lipid, polysaccharide, and terpenoid contents further illustrate the compositional diversity of these fungal materials. Reported values for *G. frondosa* include 70.70% carbohydrate and 90.10% moisture. Crude fat contents of 30.01% and 10.23% have been reported for *T. camphoratus* and *G. lucidum*, respectively. The reported crude polysaccharide contents of *P. cocos*, *G. lucidum*, and *A. heimuer* are 62.04%, 8.56%, and 4.36%, respectively. In addition, *T. camphoratus* contains 8.05% total triterpenoids, while ganoderic acid A has been reported at 655.39 μg/g in *G. lucidum*. Fungal polysaccharides have been investigated in preclinical models for their potential effects on obesity-related metabolism, anxiety-like behavior, inflammation, intestinal barrier function, and gut microbial pathways [60,61,62]. However, these effects depend on polysaccharide structure, dose, bioavailability, and experimental context.

Mineral profiles also differ among the reported fungal materials. Reported mineral values include 1953 μg/L Ca and 593.73 μg/L Fe for *P. cocos*, and 1669.42 mg/kg Ca, 956.38 mg/kg Fe, and 618.67 mg/kg Mg for *G. lucidum*. The reported K and Na contents are 1561.43 mg/kg and 62.13 mg/kg, respectively, in *A. heimuer*, and 7716 mg/kg and 2129 mg/kg, respectively, in *T. camphoratus*. Cross-sectional population studies have reported associations between the intake of selected minerals and depression [63]. In addition, a general-population study using 24-h urinary potassium excretion as an indicator of potassium intake found that lower potassium excretion was associated with a higher prevalence of anxiety, depression, and their co-occurrence [64].

Nucleosides, nucleotides, and other characteristic metabolites constitute another compositionally diverse group in edible and medicinal mushrooms. *G. lucidum* contains 1581.68 μg/g guanosine, *T. camphoratus* contains 70,000 mg/kg guanylic acid, and *O. sinensis* contains 9.02% mannitol. These compounds contribute to the compositional diversity of the fungal materials, although their physiological relevance depends on dose, bioavailability, metabolism, and the form of the final food product. More broadly, edible and medicinal mushrooms and their bioactive components have been investigated for their potential roles in obesity-related metabolic regulation and functional-food development [65].

### 4.3. Major Bioactive Components with Potential Relevance to Obesity–Depression Comorbidity

Edible and medicinal mushrooms contain diverse bioactive components, including polysaccharides, triterpenoids, nucleosides, sterols, melanins, and aromatic compounds (Table 2). In experimental models, these components were associated with the regulation of lipid and glucose metabolism, oxidative stress, inflammation, intestinal barrier function, gut microbial composition, and neurobehavioral processes. Polysaccharides from *G. lucidum*, *C. militaris*, *G. frondosa*, *A. heimuer*, and *P. cocos* were reported to improve inflammatory responses, gut microbiota-related alterations, or depressive-like behaviors in rodents. Other representative findings included the regulation of metabolism and neuroinflammation by *T. camphoratus* and *H. erinaceus* preparations, the antidepressant-like effects of *O. sinensis* polysaccharides and *C. militaris*-derived cordycepin, and the modulation of lipid accumulation, oxidative stress, and intestinal microbiota by A. heimuer melanin.

Ganoderic acid A, antroquinonol, antrocin, erinacine A, cordycepin, and pachymic acid were selected as representative small-molecule candidates for subsequent target-network analysis. Experimental studies linked these compounds to signaling systems involved in lipid metabolism, glucose homeostasis, oxidative stress, inflammation, and neural function, while network analysis was used to further explore their potential shared targets and pathways in obesity and depression. Overall, the available findings support the nutritional and functional potential of edible and medicinal mushrooms, with current evidence derived mainly from cellular and animal studies and limited preliminary observations in humans.

### 4.4. Proposed Biosynthetic Pathways of Major Bioactive Components

**Figure 3:**
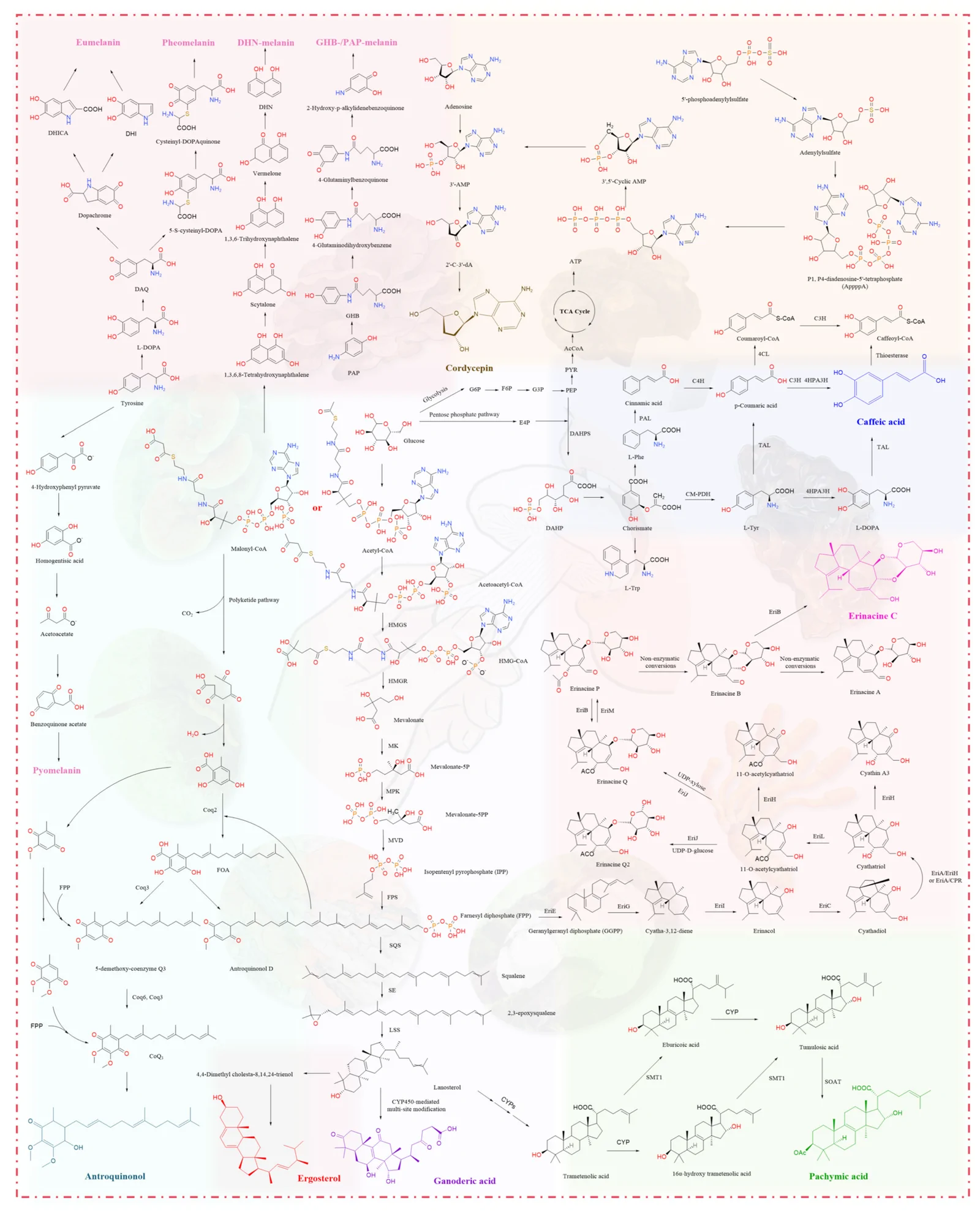
Proposed integrated biosynthetic pathways of representative bioactive components in edible and medicinal mushrooms. This original schematic was created by the authors based on information reported in Refs. [96,97,98,99,100,101,102,103].

Core bioactive components of edible and medicinal mushrooms with reported anti-obesity and antidepressant-like activities (including terpenoids, nucleosides, sterols, melanins, and phenolic acids) are connected through glucose-derived central carbon metabolism. Following carbon flux partitioning, these metabolites are biosynthesized through multiple interconnected secondary metabolic pathways, forming a structurally diverse library of bioactive compounds. Glucose is catabolized through glycolysis, the pentose phosphate pathway (PPP), and the tricarboxylic acid (TCA) cycle, supplying acetyl coenzyme A (acetyl-CoA), erythrose-4-phosphate (E4P), phosphoenolpyruvate (PEP), and ribose-phosphate derivatives. Terpenoids and sterols (e.g., ganoderic acid, erinacines, ergosterol, and pachymic acid) are mainly produced via the mevalonate (MVA) pathway: acetyl-CoA is converted into isopentenyl diphosphate (IPP) and dimethylallyl diphosphate (DMAPP) through a series of enzymatic reactions, which further condense to form farnesyl diphosphate (FPP). As a key branching node, FPP is converted through squalene and lanosterol to generate ergosterol and triterpenoids such as ganoderic acid and pachymic acid. Meanwhile, FPP is further elongated to geranylgeranyl diphosphate (GGPP) for the synthesis of diterpenoids including erinacines, whereas an FPP-derived polyprenyl side chain contributes to antroquinonol biosynthesis through a ubiquinone-related pathway. Nucleosides such as cordycepin are synthesized through purine metabolism involving phosphorylation and deoxygenation of adenosine-related intermediates. Phenolic acids (e.g., caffeic acid) are synthesized via the shikimate pathway, using E4P derived from the PPP and PEP derived from glycolysis as precursors, followed by stepwise transformation via tyrosine (Tyr) and L-3,4-dihydroxyphenylalanine (L-DOPA). Mushroom melanins are synthesized through several parallel branches: Tyr- and L-DOPA-derived metabolites form eumelanin and pheomelanin; acetyl-CoA and malonyl-CoA participate in 1,8-dihydroxynaphthalene (DHN)-melanin biosynthesis; homogentisic acid generated during Tyr degradation forms pyomelanin; and chorismate-derived metabolites contribute to γ-L-glutaminyl-4-hydroxybenzene (GHB)/p-aminophenol (PAP)-melanin biosynthesis. Collectively, the biosynthetic pathways of various bioactive components are interconnected with shared precursors, which contribute to the types and abundances of bioactive compounds in edible and medicinal mushrooms. This biosynthetic diversity provides a biochemical basis for investigating the multi-component and multi-target regulatory potential of edible and medicinal mushrooms in obesity–depression comorbidity (Figure 3).

### 4.5. Predicted Target Associations of Bioactive Components in Obesity–Depression Comorbidity

To explore potential molecular associations between representative mushroom-derived bioactive components and obesity–depression comorbidity, target-set intersections and protein–protein interaction (PPI) network analyses were performed for ganoderic acid A, antroquinonol, antrocin, erinacine A, cordycepin, and pachymic acid.

#### 4.5.1. Target-Set Intersections and Network Construction

**Figure 4:**
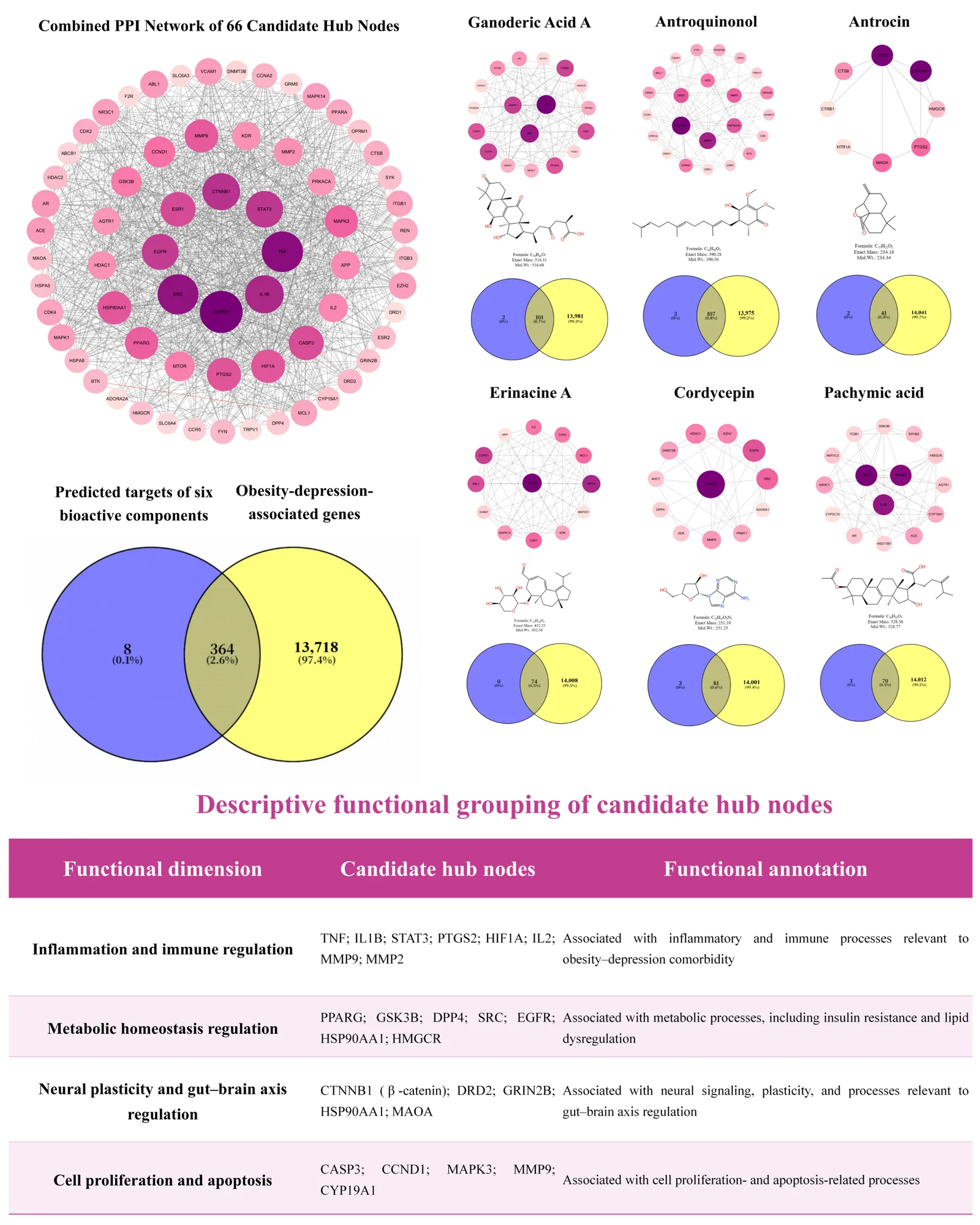
Target-set intersections, STRING-derived protein–protein association networks, and descriptive functional grouping of candidate hub nodes for six representative mushroom-derived bioactive components. **Left**: Combined PPI network of 66 candidate hub nodes and Venn diagram showing the intersection between the non-redundant union of 372 compound-associated predicted targets and the GeneCards-derived obesity–depression-associated gene set containing 14,082 genes. **Right**: Compound-specific PPI subnetworks, chemical structures, and Venn diagrams showing the intersections between the predicted targets of each component and the obesity–depression-associated gene set. In all Venn diagrams, blue represents compound-associated predicted targets, yellow represents obesity–depression-associated genes, and the overlapping region represents their intersection. In the PPI networks, node size and color intensity increase with degree, and edges represent STRING protein associations. Bottom: Descriptive grouping of representative candidate hub nodes into four functional dimensions. This grouping is interpretive and does not represent a formal functional enrichment analysis.

SwissTargetPrediction yielded 103, 110, 43, 74, 84, and 73 candidate molecular targets for ganoderic acid A, antroquinonol, antrocin, erinacine A, cordycepin, and pachymic acid, respectively. Merging these six target sets and removing duplicates produced 372 unique compound-associated candidate targets. Separately, 14,082 genes were shared between the obesity- and depression-associated gene sets retrieved from GeneCards after gene-symbol standardization and removal of duplicate and unmapped records. Intersection analysis identified 101, 107, 41, 74, 81, and 70 comorbidity-associated candidate targets for the six respective compounds. Of the 372 unique compound-associated candidate targets, 364 overlapped with the 14,082-gene obesity–depression-associated set (Figure 4).

Following the STRING and Cytoscape procedures described in Section 2.2, topological screening retained 18 nodes and 105 edges for ganoderic acid A, 23 nodes and 90 edges for antroquinonol, 8 nodes and 12 edges for antrocin, 13 nodes and 63 edges for erinacine A, 12 nodes and 33 edges for cordycepin, and 15 nodes and 52 edges for pachymic acid. The combined network contained 66 candidate hub nodes connected by 1069 STRING protein associations. GAPDH, TNF, SRC, IL1B, and CTNNB1 were among the nodes with the highest degree, betweenness centrality, and closeness centrality values in the combined network (Figure 4).

#### 4.5.2. Functional Interpretation of Candidate Hub Nodes

For descriptive interpretation, representative candidate hub nodes were organized into four functional dimensions: inflammation and immune regulation, metabolic homeostasis, neural plasticity and gut–brain axis regulation, and cell proliferation and apoptosis (Figure 4). The first dimension included TNF, IL1B, STAT3, PTGS2, HIF1A, IL2, MMP9, and MMP2, whereas PPARG, GSK3B, DPP4, SRC, EGFR, HSP90AA1, and HMGCR were associated primarily with metabolic regulation. CTNNB1, DRD2, GRIN2B, HSP90AA1, and MAOA were grouped according to their relevance to neural signaling and gut–brain axis-related processes, while CASP3, CCND1, MAPK3, MMP9, and CYP19A1 were associated with cell proliferation and apoptosis. These overlapping functional associations indicate potential convergence of the six compounds on pathways relevant to metabolic, inflammatory, and neurobehavioral regulation. These network-derived associations are hypothesis-generating and do not establish direct compound-target binding, therapeutic efficacy, or pharmacological synergy.

## 5. Regulation of the Gut–Brain Axis in Obesity–Depression Comorbidity

### 5.1. Regulatory Mechanisms

The gut–brain axis comprises bidirectional communication between the gastrointestinal tract and the central nervous system (CNS) through neural, neuroendocrine, microbiota-mediated, and immune pathways. Dysregulation of these interconnected routes has been associated with both obesity and depression and may contribute to their comorbidity. Bioactive components derived from edible and medicinal mushrooms may modulate processes within this axis, including gut microbial composition, intestinal barrier function, inflammatory signaling, and neuroendocrine regulation. For clarity, these interactions are discussed here in terms of four interrelated routes: hormonal signaling, direct neural signaling, microbiota-mediated signaling, and immune-mediated regulation [104].

#### 5.1.1. Hormonal Regulation

**Figure 5:**
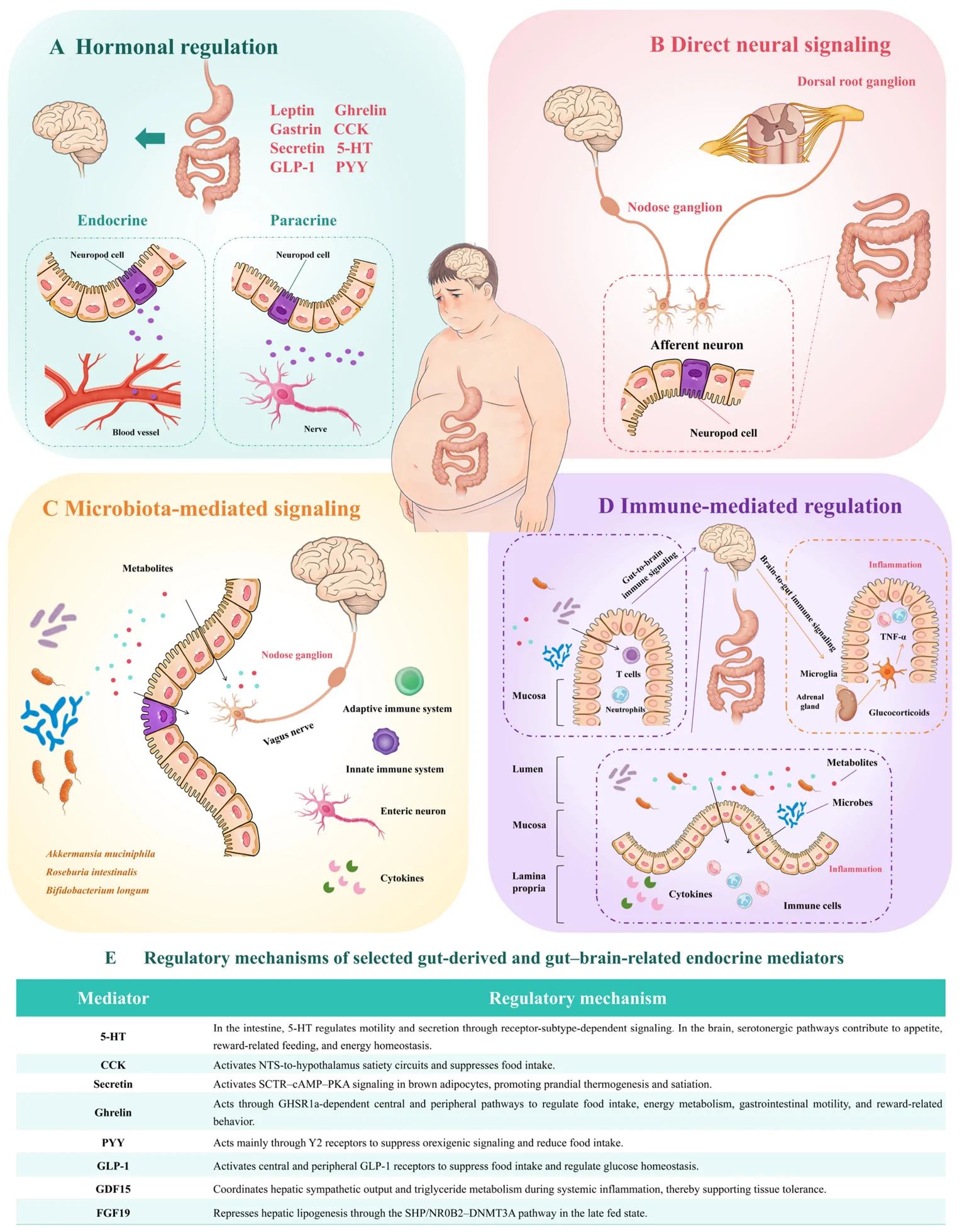
Schematic overview of major gut–brain axis signaling pathways and selected endocrine mediators relevant to obesity–depression comorbidity. (**A**) Hormonal regulation through endocrine and local signaling; (**B**) direct neural signaling through vagal and spinal afferent pathways; (**C**) microbiota-mediated signaling involving microbial metabolites, enteric and vagal pathways, and immune mediators; (**D**) bidirectional immune-mediated signaling between the gut and brain; and (**E**) reported regulatory roles of selected gut-derived and gut–brain-related endocrine mediators. The mechanisms summarized in panel E are supported by the corresponding references: 5-HT [109], CCK [110], secretin [111], ghrelin [112], PYY and GLP-1 [105], GDF15 [107], and FGF19 [108]. This original schematic was created by the authors based on evidence summarized from the cited literature.

Gut hormones are secreted predominantly by enteroendocrine cells (EECs), although enterocytes and other intestinal epithelial cell populations also contribute to gut-derived endocrine signaling [105]. EEC-derived mediators considered here include 5-hydroxytryptamine (5-HT), cholecystokinin (CCK), secretin, ghrelin, peptide YY (PYY), and glucagon-like peptide-1 (GLP-1); several of these mediators may be co-expressed within EEC lineages [105,106]. Growth differentiation factor 15 (GDF15) and enterocyte-derived fibroblast growth factor 19 (FGF19) are additionally included as metabolically relevant endocrine mediators [107,108]. EECs sense nutritional and non-nutritional stimuli through transporters, ion channels, and receptors, converting luminal signals into hormone secretion. Together, these mediators participate in the regulation of gastrointestinal, adipose-tissue, and CNS functions relevant to metabolic homeostasis (Figure 5A,E).

#### 5.1.2. Direct Neural Signaling

The vagus nerve (VN) and spinal afferent pathways provide rapid neural routes for gut–brain communication. Luminal nutrients and microbial metabolites can modulate afferent activity through epithelial and enteroendocrine signaling [113]. Some EECs are electrically excitable and form synapse-like contacts with sensory nerve fibers, enabling rapid transmission of gut-derived signals to the nervous system [104] (Figure 5B).

#### 5.1.3. Microbiota-Mediated Signaling

The gut microbiota communicates bidirectionally with the brain through the microbiota–gut–brain axis, while diet can influence microbial composition and function and thereby shape signaling relevant to cognition and emotional regulation [114]. In an 8-week randomized controlled trial involving overweight adults, viable *Akkermansia muciniphila* PROBIO and its postbiotic preparation were associated with reduced body weight and improvements in liver function, lipid metabolism, emotional well-being, and gut microbiota composition [115]. Mechanistic evidence from individuals with depression and mouse models further identified reduced abundances of *Bifidobacterium longum* and *Roseburia intestinalis*, together with reduced homovanillic acid (HVA). *R. intestinalis* did not directly produce HVA but increased the abundance of *B. longum*, thereby supporting HVA generation. Administration of HVA or either bacterium improved depression-like behaviors in mouse models, with HVA-associated preservation of hippocampal synaptic integrity supporting a microbiota–metabolite–brain signaling pathway [116] (Figure 5C).

#### 5.1.4. Immune-Mediated Regulation

The immune system constitutes a major route of microbiota–gut–brain communication. Microbial metabolites and gut-derived immune signals can shape peripheral innate and adaptive immune responses, which may subsequently affect blood–brain barrier integrity, microglial states, and neuroinflammation. Adaptive immune pathways, including the balance between regulatory T cells (Treg) and T helper 17 cells (Th17), contribute to microbial tolerance and inflammatory regulation, whereas neutrophils and other innate immune cells may participate under specific inflammatory conditions. Early life represents a critical window during which microbiota exposure shapes immune maturation. Germ-free mice and mice exposed to antibiotics early in life exhibit immune, microbial, and neurophysiological alterations that may persist into later developmental stages, although their implications for human neuropsychiatric outcomes remain to be established [117,118] (Figure 5D).

### 5.2. Bidirectional Links Between the Gut–Brain Axis and Obesity–Depression Comorbidity

Obesity–gut–brain axis—depression: Chronic HFD exposure may contribute to metabolic and behavioral disturbances through gut microbial dysbiosis, impaired intestinal barrier function, and low-grade inflammation. In HFD-fed male mice, treatment with an oleoylethanolamide-producing *Lactobacillus paracasei* F19 strain improved intestinal permeability, gut microbial composition, metabolic abnormalities, and behavioral disturbances, supporting a microbiota–gut–brain link under diet-induced obesity [119]. In another HFD-fed mouse study, *WenDan Decoction*, a multi-herb traditional Chinese medicine preparation containing *P. cocos*, restored intestinal microbial homeostasis, regulated arachidonic acid metabolism, suppressed PI3K/Akt/mTOR signaling, restored hippocampal autophagic flux, and improved depressive-like behaviors [120].

Depression–gut–brain axis—obesity: Human observational studies have associated gut microbial profiles with major depressive disorder (MDD) severity. Compared with healthy controls, individuals with moderate or severe MDD showed increased *Bacteroides* abundance, whereas depletion of *Ruminococcus* and *Eubacterium* was more evident in severe MDD [121]. Independent population-level analyses found that *Coprococcus* and *Dialister* were depleted in individuals with depression, whereas higher abundances of the butyrate-producing genera *Faecalibacterium* and *Coprococcus* were associated with better quality-of-life indicators [122]. In experimental overeating models and patients with bulimia nervosa, alterations in intestinal microbiota were linked to excessive intake of palatable foods through vagal gut–brain signaling, suggesting that microbiota-related mechanisms may contribute to pathological overeating and subsequent metabolic dysfunction [123]. More broadly, gut dysbiosis may interact with immune signaling, microbial neuroactive metabolites, the hypothalamic–pituitary–adrenal axis, the autonomic nervous system, and the enteric nervous system in depression-related gut–brain axis dysfunction [124]. Collectively, these findings support bidirectional associations among metabolic dysfunction, depressive states, gut microbial dysbiosis, and altered eating behavior.

### 5.3. Preclinical Evidence for Gut–Brain-Axis-Related Effects of Bioactive Components from Edible and Medicinal Mushrooms

Current evidence concerning the gut–brain-axis-related effects of bioactive components from edible and medicinal mushrooms is derived predominantly from animal studies. Mushroom-derived polysaccharides, triterpenoids, nucleosides, and sterols have been associated with changes in gut microbial composition, intestinal barrier function, microbial metabolites, inflammation, lipid metabolism, and neurobehavioral outcomes. In HFD-fed mice, polysaccharides from sporoderm-broken spores of *G. lucidum* attenuated obesity, hyperlipidemia, inflammation, and fat accumulation while improving gut microbial dysbiosis, short-chain fatty acid production, and intestinal barrier function [76]. In HFD-fed mice, ergosterol derived from *A. heimuer* attenuated obesity and cognitive impairment and was associated with changes in gut microbial composition [125].

Evidence related to depressive-like behavior has been reported for several other representative components. In reserpine-treated mice, triterpenoids from *T. camphoratus* improved depressive-like behavioral measures, reduced IL-6 and TNF-α levels, increased dopamine and 5-HT levels, and altered gut microbial composition [80]. In stressed mice, cordycepin produced dose-dependent changes in anxiety- and depression-like behavior, gut microbiota, serum metabolites, cytokines, 5-hydroxyindoleacetic acid (5-HIAA) levels, and adipose-tissue remodeling [126]. A water-insoluble β-1,3-glucan from *P. cocos* improved depressive-like behavior and abnormalities in neurotransmitters and inflammatory factors in CUMS-induced rats, together with changes in gut microbiota, short-chain fatty acids, and tryptophan metabolites [94]. Similarly, the CSWP-2 polysaccharide fraction from O. sinensis improved depressive-like behavioral measures in chronic-restraint-stress mice and was associated with modulation of gut microbiota, microbial metabolism, and systemic immune status [85]. Collectively, these studies provide preclinical evidence supporting further investigation of edible and medicinal mushroom components in the context of obesity–depression comorbidity.

## 6. Functional Foods, Nutraceuticals, and Mushroom-Derived Medicinal Products

**Figure 6:**
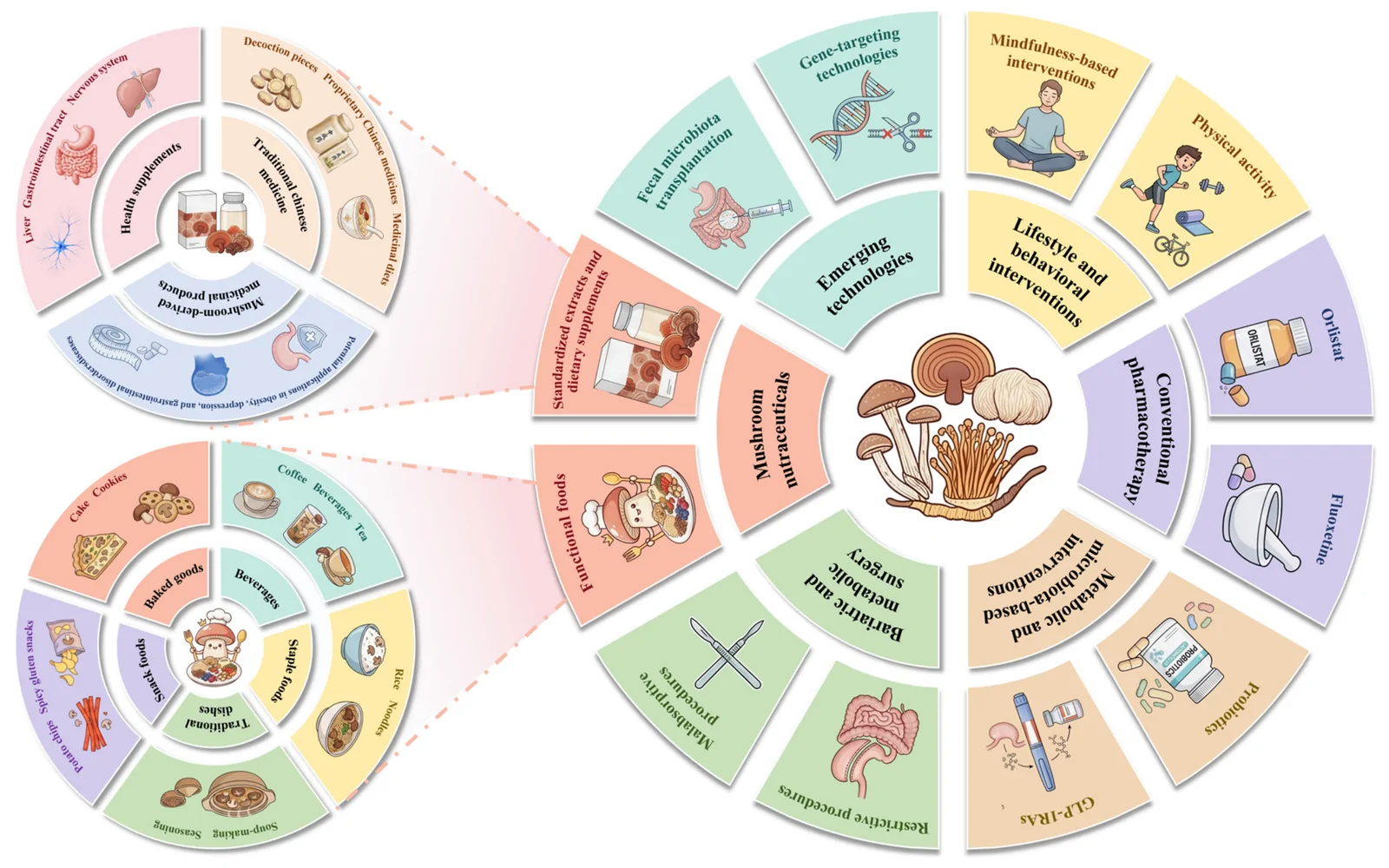
Conceptual framework of food, nutraceutical, and medicinal product categories derived from edible and medicinal mushrooms and comparative management approaches relevant to obesity–depression comorbidity.

Edible and medicinal mushrooms can be consumed as conventional foods or developed into functional foods, nutraceuticals, standardized extracts, dietary supplements, and mushroom-derived medicinal products (Figure 6). In particular, mushroom-based dietary supplements and standardized preparations provide convenient forms for incorporating mushroom-derived nutrients and bioactive components into long-term dietary management and may complement established dietary, behavioral, and medical approaches to metabolic and mental health management. Their functional potential is associated with dietary fiber, polysaccharides, triterpenoids, nucleosides, sterols, peptides, minerals, and other constituents [127]. Preclinical studies further suggest that selected mushroom preparations may modulate gut microbial composition, support intestinal barrier function, and influence inflammatory signaling and lipid metabolism [76], providing plausible links to microbiota–gut–brain axis communication.

### 6.1. Food-Based Products and Functional Food Applications

From a food-application perspective, edible and medicinal mushrooms can be consumed directly or incorporated into soups, staple foods, beverages, baked products, snacks, and seasonings as fresh mushrooms, powders, extracts, or fermented ingredients. These formats provide practical food matrices for delivering dietary fiber and mushroom-derived bioactive components, including polysaccharides, triterpenoids, nucleosides, sterols, peptides, minerals, and phenolic compounds [128]. Processing and formulation methods, including drying, extraction, and fermentation, can influence the retention, stability, bioaccessibility, and sensory properties of these constituents; their effects depend on the mushroom material, processing conditions, and final food matrix [129]. Accordingly, mushroom-based functional foods may serve as food-based or supplementary dietary approaches that complement established dietary, behavioral, and medical management, but they should not be presented as established therapies for obesity–depression comorbidity. Future development should prioritize compositional standardization, sensory acceptability, bioavailability, safety assessment, dose definition, and well-designed human intervention studies.

### 6.2. Nutraceuticals, Dietary Supplements, and Mushroom-Derived Medicinal Products

Mushroom-containing medicinal products, dietary supplements, and traditional Chinese medicine formulations provide representative examples of how edible and medicinal mushrooms have been incorporated into practical health-related applications. As summarized in Table 3, these products include approved medicinal products, commercial mushroom-based supplements, and traditional multi-herbal formulations. Although they differ in composition, regulatory status, and intended use, they collectively reflect the diverse applications of mushroom-derived ingredients in health-related products. These diverse product forms provide a foundation for exploring how mushroom-derived ingredients may contribute to metabolic regulation, gastrointestinal function, and neurobehavioral health, thereby offering new perspectives for their potential application in obesity–depression comorbidity.

#### 6.2.1. Approved Medicinal Products Containing Mushroom-Derived Ingredients

Approved medicinal products containing mushroom-derived ingredients represent one of the most established forms of mushroom utilization. As summarized in Table 3, several mushroom-based formulations have been approved and are commercially available, including *Ganoderma*-containing products (e.g., Jiawei *Ganoderma* Tablets and *Ganoderma* Safflower Tranquilizing Oral Liquid), *H. erinaceus*-based products (Leishi *Hericium* Tablets), and preparations derived from *O. sinensis*, *G. frondosa*, and *A. heimuer*. These products are mainly used for cardiovascular support, gastrointestinal discomfort, fatigue-related symptoms, and traditional deficiency-associated conditions according to their labeled indications. Beyond their traditional applications, the biological activities of mushroom-derived components provide potential links to metabolic and neurobehavioral regulation. For example, polysaccharides and triterpenoids from *Ganoderma* species have been widely investigated for immunomodulatory, antioxidant, and gut microbiota-related effects, whereas *H. erinaceus*-derived polysaccharides and erinacines have been investigated for their potential roles in gastrointestinal protection and neuroactive pathways. These mechanisms, including intestinal homeostasis, inflammation regulation, and gut–brain axis modulation, provide a mechanistic perspective for understanding the potential contribution of mushroom-containing products to obesity–depression comorbidity [130].

#### 6.2.2. Commercial Dietary Supplements and Mushroom Preparations

Commercial dietary supplements and mushroom preparations represent an important approach for incorporating mushroom-derived bioactive components into functional health products. As summarized in Table 3, commercially available formulations containing *H. erinaceus*, *Cordyceps*, and *T. camphoratus* are primarily positioned for gastrointestinal support, energy maintenance, immune health, and general vitality. Among these products, *H. erinaceus*-containing supplements have received increasing scientific interest due to their diverse bioactive constituents, including polysaccharides, erinacines, and hericenones. Experimental studies have suggested that H. erinaceus-derived components may contribute to gastrointestinal homeostasis through modulation of gut microbiota, intestinal barrier integrity, and inflammatory responses [131], while erinacines and related compounds have been investigated for their potential neuroactive effects involving neurotrophic pathways and gut–brain communication [132]. Similarly, *Cordyceps-* and *T. camphoratus*-derived preparations contain characteristic metabolites that have been investigated for immunomodulatory, antioxidant, and metabolic regulatory activities. These findings provide a mechanistic basis for considering mushroom-based supplements as potential contributors to metabolic and emotional health maintenance, while their potential contribution to obesity–depression comorbidity remains an area of growing research interest.

#### 6.2.3. Traditional Chinese Medicine Formulations Containing Mushroom-Derived Ingredients

Traditional Chinese medicine (TCM) formulations containing mushroom-derived ingredients represent another important form of mushroom utilization, particularly within classical multi-herb prescriptions. As summarized in Table 3, representative formulations such as Si Jun Zi Decoction and Shenling Baizhu Powder contain *P. cocos* together with other medicinal herbs and have been traditionally used for gastrointestinal discomfort, spleen deficiency-related symptoms, and disorders associated with impaired digestive function. Among these formulations, *P. cocos* is considered an important medicinal component due to its polysaccharides and triterpenoids, which have been investigated for immunomodulatory, anti-inflammatory, and gastrointestinal regulatory activities. Unlike single-ingredient products, TCM formulations are characterized by multi-component and multi-target interactions, where the overall biological effects arise from the combined actions of multiple herbal constituents. Modern pharmacological studies of Shenling Baizhu Powder have suggested potential roles in regulating gut microbiota, intestinal inflammation, and metabolic homeostasis, providing a framework for further understanding the potential relevance of mushroom-containing traditional formulations to metabolic and emotional health [133].

However, current strategies for obesity–depression comorbidity management mainly rely on pharmacological, surgical, lifestyle, and psychological interventions targeting metabolic and behavioral outcomes. Although these approaches have demonstrated clinical benefits, their effectiveness may still be influenced by individual variability, long-term adherence, adverse effects, and the complexity of managing interconnected metabolic and psychological alterations [134]. Emerging strategies, including microbiota-based interventions and other precision approaches targeting metabolic and neurobehavioral pathways, provide new perspectives for understanding and managing obesity–depression comorbidity [135].

In this context, mushroom-derived products represent a complementary and promising nutritional strategy due to their multi-component and multi-target characteristics. As illustrated in Figure 6, mushroom nutraceuticals may contribute to the modulation of multiple biological processes associated with obesity–depression comorbidity. Future studies integrating standardized mushroom preparations, mechanistic investigations, and clinical evaluation will be essential to clarify their role in the prevention and supportive management of obesity–depression comorbidity.

## 7. Conclusions and Perspectives

Obesity–depression comorbidity is a complex condition characterized by interconnected metabolic dysfunction, neuropsychological disturbances, chronic low-grade inflammation, gut microbiota dysbiosis, and impaired gut–brain axis communication. This review highlights the potential of edible and medicinal mushrooms as functional food and nutraceutical resources, integrating current knowledge on their cultivation and fermentation strategies, nutritional characteristics, bioactive components, and potential biological functions. Mushroom-derived compounds may contribute to the modulation of biological processes associated with metabolic and mental health through complementary mechanisms. Collectively, current evidence supports the further development of edible and medicinal mushrooms as supportive functional foods and nutraceuticals for obesity–depression comorbidity, rather than replacements for established clinical therapies.

From a functional food perspective, mushroom-based products can be developed in diverse forms, including mushroom powders, fermented products, beverages, staple foods, baked foods, snacks, nutraceuticals, standardized extracts, and mushroom-derived health products. These products provide practical approaches for incorporating mushroom-derived nutrients and bioactive components into dietary strategies. However, current evidence is still insufficient to fully establish their clinical efficacy, as most mechanistic findings are derived from in vitro studies and animal models, while human intervention studies specifically addressing obesity–depression comorbidity remain limited. In addition, variability among mushroom species and cultivation conditions, uncertain bioavailability, limited pharmacokinetic information, and insufficient product standardization remain major challenges for clinical translation.

Future studies should focus on the standardized identification and quantification of key bioactive markers, dose–response relationships, bioavailability, safety evaluation, and long-term efficacy of mushroom-derived functional products. Well-designed randomized controlled trials, multi-omics approaches, and microbiome-centered mechanistic studies will be essential to clarify how mushroom-derived components influence metabolic and neuropsychological outcomes through the gut–brain axis. Integrating food science, nutrition, microbiome research, and clinical evaluation will further support the development of evidence-based mushroom-derived functional foods and nutraceuticals for metabolic and mental health applications.

## Figures and Tables

**Table 1:** Representative production modes and reported culture conditions for selected edible and medicinal mushrooms.

Scientific Name	Production Modes			Representative Culture Conditions (Temperature, Relative Humidity or Substrate Moisture, pH, and Light)
	Cultivation and Resource Production	Fermentation-Based Production		
		SSF	LSF	
*Ganoderma lucidum*	Log cultivation, sterilized short-log cultivation, bottle cultivation, bag cultivation, casing soil cultivation, industrial cultivation [23]	Bidirectional solid-state fermentation of highland barley increased the triterpenoid content by 76.57%, while reducing hardness and chewiness by 77.96% and 58.60%, respectively [24].	Adding 0.25% Tween 80 increased mycelial biomass by 19.76%, boosted exopolysaccharide (EPS) yield by 137.50%, significantly enhanced the antioxidant activity and uronic acid content of EPS, and simultaneously decreased its molecular weight [25].	Temperature: 25–30 °C (mycelium), 22–28 °C (fruiting body) Humidity: 65% (mycelium), 90% (fruiting body) pH: 5.0–6.5 Light: 300 lx diffused light irradiation [26,27]
*Taiwanofungus camphoratus*	Log cultivation (camphor tree logs, apple tree logs, oak logs) [28]	Maltose 31.89 g/L, beef extract 6.96 g/L, KH_2_PO_4_ 1.48 g/L, MgSO_4_ 0.5 g/L, vitamin B1 0.1 g/L, and agar powder 18 g/L increased the total triterpenoid yield by 26.1% [29].	Exogenous addition of herbal medicines, aqueous/ethanol extracts of camphor tree materials, and plant hormones can increase the contents of various bioactive components (e.g., triterpenoids and 4-acetylantroquinonol B) by 1.43–12.81 times the corresponding levels [30].	Temperature: 28 °C Humidity: 50% pH: unadjusted Light: 300 lx red light [31,32].
*Hericium erinaceus*	Bottle cultivation, bag cultivation, casing soil cultivation, industrial cultivation [33]	Fermentation of olive waste achieved a biological efficiency of 31%, with a significant increase in bioactive components, and simultaneously accomplished wastewater decolorization (65%) and phenol reduction (47%) [34].	Acid-pretreated brewer’s spent grain and wheat bran achieved biological conversion rates of 38.6% and 34.8%, respectively. Secondary fermentation enabled the efficient synthesis of the secondary metabolite erinacine C, with yields of 174.8 mg/g and 99.3 mg/g, respectively [35].	Temperature: 21–25 °C (mycelium), 14–18 °C (fruiting body) Humidity: 65–70% (mycelium), 90–95% (fruiting body) pH: 5.5–6.8 Light: 50–100 lx [36]
*Ophiocordyceps sinensis*	Field semi-artificial cultivation, rice medium, artificial rearing of the host Hepialus moth, infection of Hepialus larvae by *O. sinensis* [37]	Peptone 1%, sucrose 2%, KH_2_PO_4_ 0.15%, MgSO_4_ 1%, yeast powder 1%, soybean cake powder 4%, and beef extract 2% increased the contents of cordycepin and mannitol by 63.9% and 37.9%, respectively [38].	The antioxidant activity peaked on day 30 of fermentation. Lipids, heterocyclic differential metabolites and key genes were up-regulated, and the antioxidant capacity was enhanced via the activation of pathways including glutathione metabolism [39].	Temperature: 18–20 °C Humidity: 40–50% pH: 5.0–6.0 Light: No less than half of the daily duration with light exposure [37,38,40].
*Cordyceps militaris*	Bottle cultivation, pot cultivation, silkworm-Cordyceps cultivation, industrial cultivation [41]	Solid-state fermentation with the complex cereal substrate consisting of rice, wheat, jowar, bajra and sugarcane bagasse yielded the highest cordycepin content [42].	Supplementation with 5 mg/L rotenone altered metabolic and biosynthetic pathways and increased cordycepin production by 316.09% under the reported LSF conditions [43].	Temperature: 24–27 °C (spore germination), 18–25 °C (mycelium), 20–23 °C (fruiting body) Humidity: 60–70% (mycelium), 80–90% (fruiting body) pH: 5.5–6.8 Light: 100–250 lx [41]
*Grifola frondosa*	Substitute cultivation, substitute cultivation with soil covering, industrial cultivation [44]	The polysaccharides obtained using wheat grains as the solid-state fermentation substrate exhibited prominent antioxidant and immunomodulatory activities [45].	Auxiliary application of a low-intensity alternating magnetic field can stimulate the expression of related genes, leading to altered mycelial morphology, increased amino acid content, and a 12% increase in biomass [46].	Temperature: 13–27 °C (mycelium), 15–28 °C (fruiting body) Humidity: 55–85% (mycelium), 70–95% (fruiting body) pH: 6 Light: 50–100 lx white light provided by light-emitting diodes (mycelium), diffused daylight (fruiting body) [47,48,49]
*Auricularia heimuer*	Log cultivation, industrial cultivation, open-field cultivation, protected cultivation [50]	Alkali-treated corn stover inoculated with mycelium showed the best growth performance, with a cellulose degradation rate of 35.38%. However, ozone treatment was more suitable for lignin degradation, leading to a lignin loss rate of 28.08% [51].	After optimization of submerged fermentation for *A. heimuer*, the melanin yield reached 2.97 g/L, representing a 2.14-fold increase [52].	Temperature: 23–25 °C Humidity: 60–65% (mycelium), 85–95% (fruiting body) pH: 5.5–7.5 Light: No light required for mycelial growth; sufficient diffused daylight for fruiting body development [53].
*Poria cocos*	Tree stump cultivation, dry log cultivation, substitute substrate cultivation [54]	Solid-state fermentation of *Astragalus membranaceus* by *P. cocos* led to a 5.16-fold increase in the content of astragaloside IV. Following in vitro digestion, the fermented products exhibited enrichment of active ingredients, along with significantly enhanced antioxidant activity and bioaccessibility [55].	Liquid-state fermentation of green tea extract with *P. cocos* for 17 h transformed its characteristic green, grassy notes into floral, jasmine-like, and mild citrus aromas while retaining most of its in vitro antioxidant activity [56].	Temperature: 23–28 °C Humidity: During mycelial growth and development, the inoculated substrate’s moisture content was 50%, soil moisture 25%, and air humidity about 70%. Then, during sclerotial growth and development, the cultivation cellar’s humidity should be kept at around 60%. Soil pH: 5.5–6.5 Light: No light required [54].

**Table 2:** Representative bioactive components from edible and medicinal mushrooms with potential relevance to obesity–depression-related pathways.

Scientific Name	Category	Representative Component or Preparation	PubChem CID	Evidence Model	Reported Effect or Functional Relevance	Reference
*G. lucidum*	Triterpenoids	Ganoderic acid A	471002	High-fat diet (HFD)-fed mice; Caco-2 cells; receptor-binding assays	Bound to and competitively inhibited intestinal farnesoid X receptor (FXR), reduced intestinal lipid absorption, and alleviated weight gain, hyperlipidemia, and hepatic lipid accumulation in mice.	[75]
	Polysaccharides	Sporoderm-broken spore polysaccharide	—	HFD-fed mice	Reduced obesity, hyperlipidemia, inflammation, and fat accumulation; improved gut microbial dysbiosis and intestinal barrier function; and inhibited adipose toll-like receptor 4 (TLR4)/myeloid differentiation primary response 88 (MyD88)/nuclear factor kappa B (NF-κB) signaling.	[76]
	Nucleoside-rich extract	Adenosine-rich extract	—	HFD-fed mice; proteomic and post-translational-modification analyses	Lowered lipid-related indices and altered proteins and acetylation/crotonylation profiles associated with lipid-metabolism pathways, including peroxisome proliferator-activated receptor (PPAR) signaling.	[77]
*T. camphoratus*	Ubiquinone derivative	Synthetic (+)-antroquinonol	24875259	Insulin-resistant cell models and enzyme assays	Activated AMP-activated protein kinase (AMPK), promoted glucose transporter type 4 (GLUT4) translocation and glucose uptake, and inhibited dipeptidyl peptidase IV (DPP-IV) activity under the reported experimental conditions.	[78]
	Sesquiterpene lactone	Antrocin	53474706	Network-pharmacology candidate; mechanistic cancer-cell study	Retained as one of the six compounds prioritized for subsequent target-network analysis. Antrocin isolated from *T. camphoratus* inhibited protein kinase B (Akt)/mechanistic target of rapamycin (mTOR)-related signaling in a mechanistic cell study.	[79]
	Triterpenoids	Total triterpenoid fraction	—	Reserpine-induced depressive-like mouse model	Improved depressive-like behavioral measures, reduced interleukin-6 (IL-6) and tumor necrosis factor-α (TNF-α), increased dopamine and serotonin, and partly restored gut microbial composition.	[80]
	Mixed extract	Ethanol extract containing polyphenols, flavonoids, triterpenoids, and adenosine	—	Amyloid-beta25-35-treated PC12 cells	Reduced amyloid-beta-induced neurotoxicity and mitochondrial apoptosis-related changes and downregulated adenosine A1 and A2A receptors in vitro.	[81]
*H. erinaceus*	Cyathane diterpenoid	Erinacine A and erinacine A-containing mycelium	10410568	Aged mice fed a high-fat/high-sucrose diet	Improved spatial-learning measures and reduced metabolic abnormalities and hippocampal inflammatory cytokine expression; nerve growth factor (NGF) and neuronal nuclei (NeuN) increases were reported for the mycelial preparation.	[82]
	Aromatic compounds	Hericenones C, D, F, and G	15658905 (hericenone C)	Ovariectomized mice and pancreatic-lipase assays	The mushroom powder reduced adipose tissue, plasma total cholesterol, and leptin; isolated hericenones inhibited pancreatic lipase in vitro, supporting reduced lipid absorption as a possible mechanism.	[83]
	Whole-mushroom preparation	Mycelium and fruiting-body preparation	—	Pilot intervention in overweight or obese adults	Was associated with improvements in selected mood, sleep, and binge-eating measures and changes in circulating pro-brain-derived neurotrophic factor (pro-BDNF).	[84]
*O. sinensis*	Polysaccharides	CSWP-2 polysaccharide fraction	—	Chronic restraint stress mouse model	Improved depressive-like behaviors and was associated with changes in gut microbiota, metabolites, and systemic immune status, supporting a microbiota–gut–brain-related mechanism.	[85]
*C. militaris*	Nucleosides	Cordycepin	6303	Chronic unpredictable mild stress (CUMS)-induced rat model	Improved depressive-like behaviors and metabolic abnormalities and modulated glycogen synthase kinase 3β (GSK3β)/beta-catenin signaling in a preclinical model.	[86]
	Polysaccharides	Selenium-rich crude polysaccharides	—	HFD-fed mice	Reduced serum triglycerides and low-density lipoprotein (LDL) cholesterol, attenuated obesity-related inflammation, and improved gut microbial dysbiosis.	[87]
*G. frondosa*	Polysaccharides	Polysaccharides	—	HFD-fed rats	Reduced serum lipids and hepatic steatosis, promoted fecal bile-acid excretion, and partially restored gut microbial composition.	[88]
	Sterols	Ergosterol peroxide	5351516	Palmitate-treated C2C12 cells	Reduced reactive oxygen species and improved glucose uptake through modulation of phosphoinositide 3-kinase (PI3K)/Akt, mitogen-activated protein kinase (MAPK), and GLUT4-related signaling in vitro.	[89]
	Flavonoid-rich extract	Ethyl-acetate extract rich in pseudobaptigenin and cyanidin-3-O-xylosylrutinoside	—	HFD-fed rats	Reduced serum glucose, triglycerides, LDL cholesterol, and hepatic fat accumulation and altered gut microbiota and glycolipid-metabolism-related gene expression.	[90]
*A. heimuer*	Polysaccharides	Polysaccharides	—	HFD-fed mice	Attenuated obesity and metabolic abnormalities through a gut-microbiota-dependent mechanism involving *Papillibacter cinnamivorans*, intestinal lipid absorption, and hepatic thermogenesis.	[91]
	Melanins	Melanin	—	Alcohol-exposed mouse model	Auricularia melanin reduced serum triglyceride, total cholesterol, and LDL cholesterol levels, attenuated hepatic lipid accumulation and oxidative stress, and modulated the intestinal microbiota in alcohol-exposed mice.	[92]
	Polysaccharide–sterol complex	Polysaccharide–ergosterol complex	—	In vitro cholesterol-micelle model	Improved the photothermal stability of ergosterol and reduced cholesterol solubility in micelles.	[93]
*P. cocos*	Polysaccharides	Water-insoluble β-1,3-glucan	—	CUMS-induced rat model	Improved depressive-like behaviors, neurotransmitter and inflammatory-factor abnormalities, and modulated gut microbiota, short-chain fatty acids, and tryptophan metabolites.	[94]
	Triterpenoids	Pachymic acid	5484385	Oleic-acid/palmitic-acid-treated primary mouse hepatocytes	Activated sirtuin 6 (SIRT6)-dependent peroxisome proliferator-activated receptor alpha (PPARα) and nuclear factor erythroid 2-related factor 2 (Nrf2) signaling, promoted fatty-acid oxidation, and reduced lipid accumulation and oxidative stress in vitro.	[95]

**Table 3:** Selected mushroom-containing medicinal products, dietary supplements, and traditional formulations: composition, product category, intended use, and source identifiers.

Product/Formulation	Composition	Product Category	Labeled/Reported Use	Source/Identifier
(A) Approved medicinal products				
Jiawei *Ganoderma* Tablets	Deep-cultured Ganoderma dry extract and other excipients.	Chinese patent medicine	“Tonifies qi and nourishes blood, calms the mind, and unblocks the collaterals.” The product label indicates use for coronary heart disease, angina pectoris, hyperlipidemia, and cardiac arrhythmia.	NMPA approval No. Z37021522
Leishi *Hericium* Tablets	*Hericium* mycelium; excipients include starch, sucrose, gelatin, talc, Sichuan wax, and magnesium stearate.	Chinese patent medicine	“Nourishes the stomach and harmonizes the middle”; indicated for stomach pain caused by chronic superficial gastritis.	NMPA approval No. Z20003332
*Ganoderma* Safflower Tranquilizing Oral Liquid	Deep-cultured *Ganoderma* extract and other herbal ingredients (safflower, hawthorn, cinnamon, clove, and *Myristica fragrans*).	Chinese patent medicine	“Strengthens the spleen, dispels dampness, harmonizes the stomach and calms the mind.” The product label indicates use for reduced appetite and insomnia associated with spleen deficiency/dampness and restlessness.	NMPA approval No. B20020694
*Ganoderma* Two-Vitamin Methionine Capsules	*Ganoderma* extract, methionine, vitamin B12, vitamin B2, and other excipients.	Compound medicinal preparation containing *Ganoderma* extract	The product label indicates use for post-illness weakness, loss of appetite, weight loss, overexertion, and neurasthenia; also used as an adjunctive treatment for coronary heart disease, chronic hepatitis, bronchial asthma, anemia, rheumatic arthritis, and gastric ulcers.	NMPA approval No. H45021393
*Ophiocordyceps* Five-Ingredient Granules	*Ophiocordyceps sinensis*, *Schisandra chinensis*, and other ingredients.	Traditional medicinal preparation	The product label indicates use for deficiency-related symptoms, including dizziness, blurred vision, fatigue, chest tightness, shortness of breath, loss of appetite, spontaneous sweating, night sweating, and lumbago associated with spleen–kidney deficiency.	NMPA approval No. B20020850
*Grifola Frondosa* Capsules	*Grifola frondosa* polysaccharides.	Chinese patent medicine	The product label indicates use for deficiency-related symptoms, including fatigue, weakness, reduced appetite, abdominal distension after meals, and use in patients with tumor-related conditions experiencing the above symptoms after radiotherapy or chemotherapy.	NMPA approval No. B20020023
Yi Zhi Ping Capsules (*Auricularia heimuer* Capsules)	*Auricularia heimuer* preparation	Chinese patent medicine	The product label indicates use for hyperlipidemia associated with spleen deficiency and phlegm stasis, with symptoms including fatigue, lassitude, dizziness, palpitations, and chest tightness.	NMPA approval No. Z19990070
(B) Commercial dietary supplements and mushroom preparations				
ALLNATURE *Hericium Erinaceus & Tremella Fuciformis & Probiotic*	*Hericium erinaceus*, *Tremella fuciformis*, *Lycium chinense*, *Poria cocos*, *Taraxacum mongolicum*, *Coicis Semen, Dioscoreae Rhizoma* and probiotic.	Nutritional product containing mushroom ingredients and probiotics.	Supports gastric and gastrointestinal health.	All Nature Pharm Group Limited
Micotherapy *Hericium*	*Hericium sporophorum* mycelium and fruiting body extract; standardized for polysaccharides, erinacine A, and ergothioneine content.	Dietary supplement.	Support for the nervous system and gastrointestinal system.	AVD Reform [84]
MRM Nutrition *Cordyceps*	Standardized *Cordyceps* extract containing cordycepic acid and adenosine	Dietary supplement.	Supports energy, immune and respiratory health, and physical endurance.	MRM Nutrition
*Taiwanofungus camphoratus* Dropping Pills	*Taiwanofungus camphoratus* fruiting body preparation	Dietary supplement/mushroom preparation	Supports general health and vitality.	Taiwan, China
(C) Traditional Chinese medicine formulations				
Si Jun Zi Decoction	*Panax ginseng*, *Atractylodes macrocephala*, *Poria cocos*, and *Glycyrrhiza uralensis*	Classical multi-ingredient traditional formula.	Traditionally used to tonify qi and strengthen the spleen. The source describes its application for spleen–stomach qi deficiency characterized by pale complexion, low voice, weakness, reduced food intake, pale tongue, and weak pulse.	Formulary of the Pharmacy Service for Benefiting the People in the Taiping Era
Shenling Baizhu Powder	*Panax ginseng*, *Atractylodes macrocephala*, *Poria cocos*, *Glycyrrhiza uralensis*, *Dioscoreae Rhizoma*, *Nelumbo nucifera (Lianzi)*, *Coicis Semen*, and additional herbal ingredients.	Classical multi-ingredient traditional formula.	Traditionally used to strengthen the spleen and replenish qi. The source describes its application for spleen deficiency with dampness-related manifestations, including reduced appetite, weakness, diarrhea, cough with excessive phlegm, and loose stools.	Formulary of the Pharmacy Service for Benefiting the People in the Taiping Era

Abbreviation: NMPA, National Medical Products Administration.

## Data Availability

The original contributions presented in this study are included in the article. Further inquiries can be directed to the corresponding authors.

## Abbreviations

The following abbreviations are used in this manuscript:

SANRA	Scale for the Assessment of Narrative Review Articles
CNKI	China National Knowledge Infrastructure
SMILES	Simplified Molecular Input Line Entry System
STRING	Search Tool for the Retrieval of Interacting Genes/Proteins
SSF	Solid-state fermentation
LSF	Liquid-state fermentation
EPS	Exopolysaccharide
HFD	High-fat diet
FXR	Farnesoid X receptor
TLR4	Toll-like receptor 4
MyD88	Myeloid differentiation primary response 88
NF-κB	Nuclear factor kappa B
PPAR	Peroxisome proliferator-activated receptor
AMPK	AMP-activated protein kinase
GLUT4	Glucose transporter type 4
DPP-IV	Dipeptidyl peptidase IV
Akt	Protein kinase B
mTOR	Mechanistic target of rapamycin
IL-6	Interleukin-6
TNF-α	Tumor necrosis factor alpha
NGF	Nerve growth factor
NeuN	Neuronal nuclei
BDNF	Brain-derived neurotrophic factor
CUMS	Chronic unpredictable mild stress
GSK3β	Glycogen synthase kinase 3 beta
LDL	Low-density lipoprotein
PI3K	Phosphoinositide 3-kinase
MAPK	Mitogen-activated protein kinase
SIRT6	Sirtuin 6
PPARα	Peroxisome proliferator-activated receptor alpha
Nrf2	Nuclear factor erythroid 2-related factor 2
PPP	Pentose phosphate pathway
TCA	Tricarboxylic acid
E4P	Erythrose-4-phosphate
PEP	Phosphoenolpyruvate
MVA	Mevalonate
IPP	Isopentenyl diphosphate
DMAPP	Dimethylallyl diphosphate
FPP	Farnesyl diphosphate
GGPP	Geranylgeranyl diphosphate
Tyr	Tyrosine
L-DOPA	L-3,4-Dihydroxyphenylalanine
DHN	1,8-Dihydroxynaphthalene
GHB	γ-L-Glutaminyl-4-hydroxybenzene
PAP	p-Aminophenol
PPI	Protein–protein interaction
CoA	Coenzyme A
CNS	Central nervous system
EECs	Enteroendocrine cells
5-HT	5-Hydroxytryptamine
CCK	Cholecystokinin
PYY	Peptide YY
GLP-1	Glucagon-like peptide-1
GDF15	Growth differentiation factor 15
FGF19	Fibroblast growth factor 19
VN	Vagus nerve
HVA	Homovanillic acid
Treg	Regulatory T cells
Th17	T helper 17 cells
NTS	Nucleus tractus solitarius
SCTR	Secretin receptor
cAMP	Cyclic adenosine monophosphate
PKA	Protein kinase A
GHSR1a	Growth hormone secretagogue receptor 1a
SHP	Small heterodimer partner
NR0B2	Nuclear receptor subfamily 0 group B member 2
DNMT3A	DNA methyltransferase 3 alpha
MDD	Major depressive disorder
5-HIAA	5-Hydroxyindoleacetic acid
GLP-1RAs	Glucagon-like peptide-1 receptor agonists
NMPA	National Medical Products Administration
TCM	Traditional Chinese medicine
